# The Different Effects of Retirement on Life Satisfaction between the Young-old and Old-old.

**DOI:** 10.1093/geroni/igaf122.3664

**Published:** 2025-12-31

**Authors:** Meeryoung Kim

**Affiliations:** Daegu University, Daegu, Republic of Korea

## Abstract

Korea’s retirement age is 60, the lowest among OECD countries. Therefore, older adults must take steps to prepare for 20-30 years after retirement. The effects of retirement can vary depending on whether it is mandatory or voluntary. According to Lowe and Kahn’s theory of successful aging and Havigust’s activity theory, active lifestyles can contribute to retirees’ life satisfaction. As Neugarten defined age differences, this study divided older adults into young-old (ages 60-79) and old-old (age 80 and older) and compared differences in retirement adjustment. This study used the Korean Retirement Income Study data(the ninth and ninth additional panel: 399 young-old and 155 old-old). Hierarchical multiple regressions were used for data analysis. Demographic variables were added in the first step, followed by retirement-related variables, and social variables were included in the third step. The findings showed that the effects of voluntary and mandatory retirement on life satisfaction were statistically significant only for young-old. Furthermore, economic independence was important for young-old. Physical health was important for both young-old and old-old. For young-old, leisure time was a significant factor influencing life satisfaction. However, social relationships were significant indicators for both groups. This implied that leisure time is crucial for the post-retirement adjustment of young-old because they often have limited opportunities to enjoy leisure time due to time constraints before retirement. Furthermore, social relationships that foster a sense of belonging are crucial regardless of age. This suggests that programs and policies that support the continued social relationships of physically frail old-old are particularly desirable.

